# Creative Arts-Based Approaches to Improve the Well-Being of Older Adults

**DOI:** 10.1093/geroni/igab046.900

**Published:** 2021-12-17

**Authors:** Darina Petrovsky, Justine Sefcik

## Abstract

As older adults age they may face cognitive impairment, disruption in their sleep, and a decrease in mood and overall well-being. Given the negative consequences of the COVID-19 pandemic, they may experience a disruption in their access to health care services. Creative arts-based approaches have shown promise in improving the well-being of older adults and may be helpful in augmenting health care services. In this symposium, we will present research results of creative arts-based interventions aimed at improving the well-being of older adults, including those with dementia. We will also discuss ways to successfully engage with organizations that serve older adults using arts-based interventions. The first presentation will focus on the results from a feasibility randomized controlled trial examining the effects of a tailored music listening intervention on sleep outcomes in older adults living with dementia and their caregivers. The second presentation will report findings from the Mason Music & Memory Initiative aiming to improve mood and behavioral outcomes in persons living with dementia in nursing homes. The third presentation will focus on the preliminary results of a pilot study that integrated the delivery of music therapy telehealth with remote social work support and service linkage for rural older adults from low-income areas. The fourth presentation will report findings from the capacity-building program for teaching artists, health/aging organizations, and arts organizations committed to strengthening Creative Aging efforts through research. Implications for future research and creative arts-based intervention development for older adults will be discussed.

